# Customizing spatial remapping of letters to aid reading in the presence of a simulated central field loss

**DOI:** 10.1167/jov.24.4.17

**Published:** 2024-04-18

**Authors:** Colin S. Flowers, Gordon E. Legge, Stephen A. Engel

**Affiliations:** 1 Department of Psychology, University of Minnesota, Minneapolis, MN, USA; 2 Center for Applied and Translational Sensory Sciences, University of Minnesota, Minneapolis, MN, USA

**Keywords:** low vision, reading, spatial remapping, letter recognition, central field loss

## Abstract

Reading is a primary concern of patients with central field loss (CFL) because it is typically performed with foveal vision. Spatial remapping offers one potential avenue to aid in reading; it entails shifting occluded letters to retinal areas where vision is functional. Here, we introduce a method of creating and testing different remapping strategies—ways to remap text—customized for CFL of different shapes. By simulating CFL in typically-sighted individuals, we tested the customization hypothesis—that the benefits of different remapping strategies will depend on the properties of the CFL. That is, remapping strategies will aid reading differentially in the presence of differently shaped CFL. In Experiment 1, letter recognition in the presence of differently shaped CFL was assessed in and around central vision. Using these letter recognition “maps” different spatial remappings were created and tested in Experiment 2 using a word recognition task. Results showed that the horizontal gap remapping, which did not remap any letters vertically, resulted in the best word recognition. Results were also consistent with the customization hypothesis; the benefits of different remappings on word recognition depended on the different CFL shapes. Although the horizontal gap remapping resulted in very good word recognition, tailoring remapping strategies to the shape of patients’ CFL may aid reading with the wide range of sizes and shapes encountered by patients with CFL.

## Introduction

Central field loss (CFL) resulting from disorders such as advanced age-related macular degeneration (AMD) affects visual perception in a portion of the central visual field. The most affected region, the scotoma*,* is defined as an area without, or with reduced, light sensitivity and is typically measured using perimetry (Schuchard, Naseer, & de Castro, 1999). The loss of central vision can be debilitating in many everyday tasks. One particularly important task is reading, which usually relies on the high acuity of central vision to recognize letters. Reading impairment is a leading contributor to a reduced quality of life in patients with AMD (Hassell, Lamoureux, & Keeffe, 2006). Because of the prevalence of and increasing rates of AMD (Friedman et al., 2004; Kawasaki et al., 2010; Li et al., 2020; Wong et al., 2014), finding methods to mitigate the effects of CFL on reading is an important concern. Here, we use simulated CFL in typically-sighted individuals to explore a method of aiding reading.

CFL can be simulated in different ways. One approach relies on using contact lenses with an occluding area, such as a black spot, over central vision (e.g., Almutleb, Bradley, Jedlicka, & Hassan, 2018; Klee, Link, Sinzinger, & Haueisen, 2018). Another method uses an eye tracker to detect the viewer's current fixation location on a screen and occludes the portion of the screen at and around the fovea in a gaze-contingent way (Crane & Kelly, 1983; Geringswald, Baumgartner, & Pollmann, 2013). The simulated scotoma is sometimes displayed as a monochromatic region to alert participants to the occlusion (e.g., Kwon, Nandy, & Tjan, 2013; Yu & Kwon, 2023), outlined to demarcate the border of the simulated scotoma for the viewer (e.g., Barraza-Bernal, Rifai, & Wahl, 2018), and other times is presented as the same color as the background without any demarcating border (e.g., Geringswald, Baumgartner, & Pollmann, 2012; Geringswald et al., 2013). The less-visible scotomata may be sometimes preferable because many patients with CFL from AMD are completely unaware of their scotomata (Fletcher, Schuchard, & Renninger, 2012) and because some visual artifacts and attentional effects may emerge with visible scotomata (Aguilar & Castet, 2011). Perceptual completion has also been found across CFL in participants with AMD (Zur & Ullman, 2003) and filling in simulated scotomata with black or gray patches has recently been criticized as failing to match the visual experience of people with CFL (Peli, Goldstein, & Jung, 2023). However, there may be utility in raising awareness of the scotoma because this may aid reading (Pratt, Stevenson, & Bedell, 2017).

In the work reported here, CFL is simulated by preventing any stimuli from being presented in an area around foveal fixation measured using an eye tracker. The area is not demarcated or filled in and blends into the background of the display. Using this method, we tested different ways of presenting letters outside of the CFL. This general technique is referred to as spatial remapping, and it entails shifting letters or words occluded by the CFL to a peripheral region where vision is spared (Calabrèse, Liu, & Legge, 2017; Gupta et al., 2018; Ho, Loshin, Barton, & Juday, 1995; Massof, Rickman, & Lalle, 1994; Wensveen, Bedell, & Loshin, 1995). In both current experiments, stimuli were presented briefly only once and while foveal fixation was confirmed to be at the center of the screen. Trials were presented in a gaze-contingent manner where trials were canceled if fixation left the center of the screen. Our highly controlled paradigms afforded viewers 200 ms to identify text, an amount of time similar to a single fixation duration during typical reading (Rayner, 1998; Rayner & McConkie, 1976). The short time course of the trials and spatial constraint of fixation position prevented oculomotor strategies and influences that are critical components of some visual tasks with CFL such as free reading (Rubin & Feely, 2009)

The current method also focuses on shifting letters to locations that best facilitate letter recognition and reading. To do this, trigram letter recognition was measured across the visual field to identify locations where letter recognition was better and worse (Experiment 1). These data informed spatial remapping by identifying which locations should be remapped and which locations would be best for the remapped letters. Different remapping strategies, each created using the trigram letter recognition maps, were tested with three different shapes of CFL using a word recognition task (Experiment 2).

The central hypothesis in this study is that it is important to identify both locations that facilitate letter recognition and locations that impede it and to tailor remapping strategies accordingly. According to this *customization hypothesis*, it is expected that relative reading performance with different remapping strategies will vary for differently shaped CFL.

## Experiment 1: Mapping trigram letter recognition

In Experiment 1, trigram letter recognition was assessed over a portion of the visual field including and surrounding central vision. Trigrams, strings of three randomly chosen letters, are often used to probe the visual span—the number of letters that can be processed in a single fixation (Legge et al., 2007; McConkie & Rayner, 1975). Letter recognition processing of trigrams share some similarities with that of words because crowding and peripheral letter placement are both present, making it a better predictor of reading than single letter acuity, for example in RSVP reading (Legge, Mansfield, & Chung, 2001) and sentence reading (Yu, Cheung, Legge, & Chung, 2007; Yu, Park, Gerold, & Legge, 2010). Because of these links with reading performance, trigram letter recognition was collected in Experiment 1 and used to create candidate remappings for Experiment 2. It is important to note that other contributions to reading performance exist that are not probed in the trigram letter recognition task, such as lexical information.

### Methods

#### Participants

Participants were eight students, staff members, faculty of University of Minnesota, or members of the local community (five females, two males, one unreported; Age mean = 31.6 years, sd = 12.6; including authors CSF and SAE). All participants reported normal or corrected-to normal vision. The eye tracker failed to calibrate for two additional study enrollees, for whom no data were collected. Data from the other eight participants were used in analyses. Procedures were approved by the UMN Institutional Review Board and were in accordance with the Helsinki Declarations.

#### Apparatus

An ASUS 24′ LCD monitor running at 100 Hz with a 1920 × 1080 pixel resolution was used for stimulus presentation. Participants viewed the screen from 90 cm (33.01° × 18.83°) with their head supported by a chinrest. The experiment was run using Matlab (The Mathworks Inc., Natick, MA, USA) with psychophysics toolbox (Kleiner et al., 2007) and Tobii SDK extensions. A live feed of the experiment screen was projected to a second monitor outside of the room that the experimenter could monitor during the experiment. Eye fixation was monitored using a Tobii TX300 (Tobii, Stockholm, Sweden) running at 120 Hz. Initial fixation calibration was performed with the Tobii eye tracker manager before the experiment and then using custom calibration scripts during the experiment.

#### Stimuli

##### Letters

On each trial, up to three lower case letters (letter trigrams; x-width = 0.74°; courier new font) were presented along horizontal rows of an invisible grid. Letters were black (0.34 cd/m^2^) and presented on a white background (231 cd/m^2^). The letters were randomly chosen, and the same letters could appear multiple times in the same trial. On a given trial, letters were presented at three adjacent letter locations along a single horizontal row. If one or more of these letters fell within the CFL (see simulated central field loss) only one or two letters may have been presented. Trials where all three letters would fall in such locations were skipped. Letter trigrams, rather than individual letters, are used to probe letter identification within a task more akin to reading involving processes such as crowding (Chung, Levi, & Legge, 2001; Yu et al., 2007).

##### Grid

**Figure 1.**
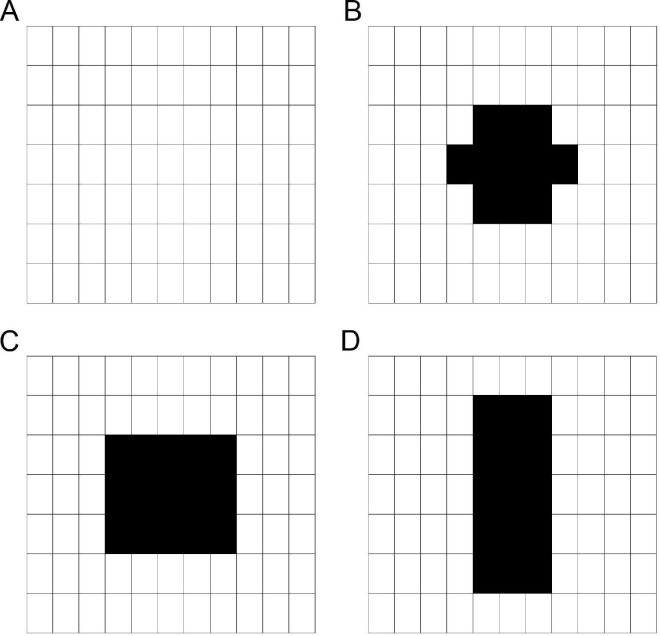
Grid (**A**) with tested simulated CFL overlaid. (**B**) “Circular,” (**C**) horizontally-elongated rectangle, and (**D**) vertically-elongated rectangle. These grids were never visible during the experiment.

Letters were presented within an invisible rectangular grid of letter locations that spanned 11.24° × 10.79° (Figure 1). The grid contained seven rows and 11 columns (77 total letter locations). Each letter location was 1.02° × 1.54°. Letters were centered at the center of each location, which allowed for a scalar letter spacing of 1.16× (see Chung, 2002) between adjacent horizontal letter locations.

##### Simulated CFL

Four conditions were tested for each participant simulating CFL with different characteristics. In the control condition, letters appeared at all possible letter locations (no simulated CFL; Figure 1 A). In the remaining conditions, letters never appeared at some central locations and letter recognition at these locations was not assessed. The three simulated CFL were “circular”, horizontally-elongated rectangle, and vertically-elongated rectangle (Figures 1B through 1D). Scotomata in patient populations vary greatly in size, shape, and continuity. Here we test a small set of relatively localized simulated CFL types to investigate how remapping strategies impact reading in the presence of differently shaped CFL. Future work can explore the impact of different sizes, scotoma opacity, or only portions of letters being occluded.

#### Procedure

Upon arriving at the laboratory, participants were given a consent form to read and sign indicating that they agreed to participate in the research approved by University of Minnesota's Human Research Protections Program. Next, they were asked to answer brief demographic questions. Participants then underwent eye tracker calibration before the experimenter read aloud the instructions that were presented on the screen. During instructions, participants viewed images of the fixation dots and sample trials with letters. They were asked if they had any questions before completing practice trials (see below) and before beginning the main experimental trials. After the experimental trials, participants were debriefed about the experiment.

##### Trial structure

In each trial, a letter location along the tested row was randomly chosen and trigrams were centered at that letter location. If all locations fell in an area of simulated CFL, a new location was chosen. Each trial started with two black fixation dots centered 0.77° above and below the center of the screen. The dots had a diameter of 0.38° and were spaced so that they did not overlap with the central letter that appeared in some trials during the experiment. The dots and letters were presented on a white background, which was present throughout the experiment. Participants were asked to fixate between the dots. Once fixation was measured (with the eye tracker) to be within 1° of the center of the screen for a full second, a trial was initiated. When fixation was further than 1° from fixation, the fixation dots turned gray, signaling to the participant to re-center their gaze between the two dots. If the trial did not initiate within 10 seconds, participants were offered the opportunity to recalibrate the eye tracker and continue.

Once the trial initiated, the letters appeared on the screen for 200 ms; the fixation dots remained on the screen to help maintain fixation. If participants’ fixation strayed beyond 1° from the center of the screen between the two dots, the trial was discarded, and participants were reminded to focus on the center of the screen. Discarded trials were replaced later in the block with new randomly selected letters. After the 200 ms letter presentation, the letters were replaced with a uniform white fill, and participants were prompted to type any letters that they saw. The response screen prompted the participants with how many letters were present on the trial, and their response should match that number of letters. Their typed response was shown on the screen, and participants could edit their responses using backspace in the event of a typo. After each trial, participants were offered an opportunity to recalibrate the eye tracker.

##### Eye tracker calibration

Eye tracker calibration consisted of a sequential five fixation point display. Four of the fixation points were placed in the four corners of the screen 3.3° horizontally from the edge of the screen and 1.9° vertically from the edge of the screen. The final fixation point was presented at the center of the screen. Each fixation point was presented for one second before the eye tracker collected fixation data for calibration. This process was repeated if the eye tracker failed to calibrate.

##### Practice trials

Participants completed practice trials for the CFL shape that was tested first. Practice (and all) trials were presented in blocks covering one row at a time, and the order of rows tested was randomized. Each block of trials was preceded by a preview screen showing which row was to be tested. During practice trials, participants completed three trials for each of the seven rows (21 trials total). During practice trials, the experimenter was in the room and available to answer any questions that the participant had. The experimenter told the participant the goal of eye tracker calibration and told them that they should recalibrate if the trials were taking a long time to initiate as characterized by the fixation dots frequently turning gray indicating poor calibration.

##### Experimental trials

The four differently shaped simulated CFL (no simulated CFL, circle, horizontally-elongated, vertically-elongated) were tested over six blocks. The four conditions were first tested in a random order, and whichever two CFL shapes were tested first were then tested again at the end. All rows of the grid for CFL were tested in a random order before the next CFL shape was tested.

Each row of the imaginary grid was tested in a block of trials for the condition being tested. The order of rows tested was randomized. Within each block, all grid locations along that row had 2 trials centered on that location with the exception of locations where all three letters would fall in an area of simulated CFL. If the leftmost and rightmost letter location was selected, letters were placed in adjacent equally spaced locations off the grid; these letter locations were not analyzed.

As a result, each letter location was probed in six letter recognition trials. This consisted of two trials where the letter was the leftmost letter out of the three letters, two trials where the letter was in the middle of the three letters, and two trials where it was the rightmost letter. There were a few exceptions to this. Only four letter recognition responses were collected for each position in the first and final column of the grid because there were never trials centered one position to the left or right of the grid. Therefore the leftmost location never had letter recognition probed when the letter was the trailing letter, and the rightmost location never had letter recognition probed when the letter was the leading letter.

Locations within two horizontal locations of simulated CFL were also probed slightly differently. These locations still had six observations per condition, but on some trials only one or two letters total were presented because some of the to-be-presented letters fell within the simulated CFL. Letters directly to the left of the simulated CFL never had letter recognition probed when it was the middle of three letters or if it were in a leading position. The inverse was true for letters directly to the right of the simulated CFL.

### Results and discussion

**Figure 2.**
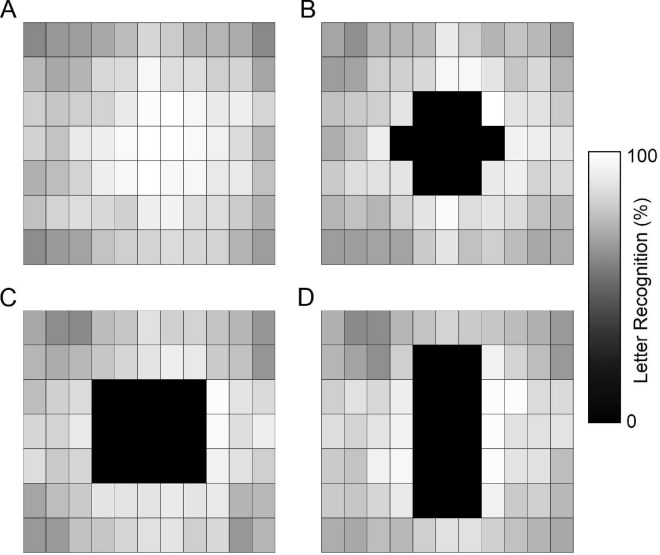
Letter recognition results from Experiment 1. CFL was not simulated in the no CFL condition (Panel **A**) and letters never appeared in the locations of the simulated CFL (**B**–**D**). The 0% performance in these locations is imputed simulating complete occlusion from CFL.

Trigram letter recognition accuracies were computed for each participant and letter position by dividing the number of correctly identified letters by the total number of trials where a letter appeared at that location (usually six trials). Letters were scored as correct only if they were reported in the correct left-to-right order. The trigram letter recognition accuracies for each of the simulated CFL shapes were averaged across participants, and these participant-averaged letter recognition scores were displayed as grayscale images, we refer to as heat maps (Figure 2). In the heat maps with simulated CFL, the areas where letters were not shown were replaced by 0% accuracy recognition to simulate a full occlusion of the letter.

The letter recognition heat maps are comparable to other studies examining letter recognition over space. The letter recognition performance of the central row is similar to that measured by tasks investigating visual span (He & Legge, 2017; Legge, Ahn, Klitz, & Luebker, 1997; Legge et al., 2001; Nazir, O'Regan, & Jacobs, 1991) or perceptual span (McConkie & Rayner, 1975). Although many of these studies focused along the horizontal meridian, the current study also probes letter recognition up to 4.5° above and below the horizontal midline.

## Experiment 2: Evaluating remapping strategies

In Experiment 2, word recognition performance was assessed with different strategies for placing letters of a word on the screen while avoiding the simulated CFL. We refer to these strategies as remappings, because they remap letters from the CFL locations to different parts of the display, and we term a specific path the letters follow from left to right across the display as a trajectory. The goal of this experiment was to test the customization hypothesis—whether utility of remapping strategies will vary with CFL shape. We used the results of Experiment 1 to tailor the remappings to locations with the best letter recognition for each CFL shape.

In typical reading, letters appear in a continuous left-to-right horizontal line of text. When letters are shifted from an area occluded by CFL to a visible area, this left-to-right horizontal continuity is necessarily interrupted. We term these breaks from typical horizontal linearity of text “unusual features,” and they include diagonals, vertical gaps, and horizontal gaps. The specific trajectories tested in Experiment 2 avoided the simulated CFL by using a combination of these unusual features. For example, a diagonal line down and to the right followed by a diagonal line up and to the right could create a V-shaped trajectory that bypasses CFL. Each trajectory was created by maximizing trigram letter recognition accuracy, but word reading is known to involve more than letter recognition. These different remapping strategies are tested to additionally explore how the features within patterns of letter placement (e.g., whether they form a continuous line or have breaks) impact word recognition beyond the underlying letter recognition accuracy.

### Remapping conditions

**Figure 3.**
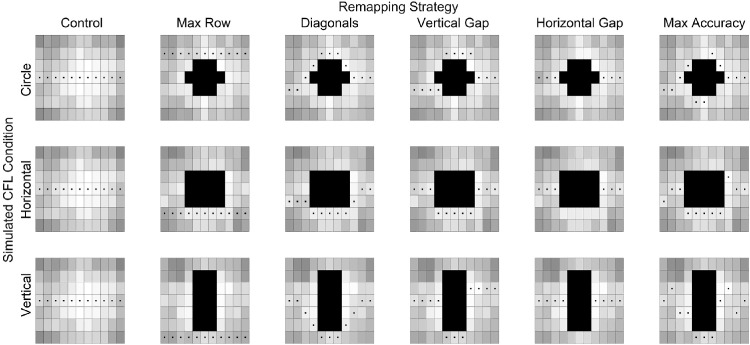
Trajectories tested in Experiment 2. Columns index the different remapping strategies, and rows CFL shape (which was tested between subjects in Experiment 2). The boxes with a centered dot represent letter locations where a letter could appear. Trajectories were created using the recursive search algorithm that used the heat maps from Experiment 1 for the given CFL shape, with the heat map shown beneath the trajectory. The control trajectory was the original horizontal letter placement without simulated CFL.

We tested five archetypal trajectories, each using a different combination of unusual features to avoid the different CFL conditions (see Figure 3). We call them archetypal because each was created for a given CFL shape using the letter perimetry data (as described below): (1) Diagonal trajectories consisted of horizontal spans of letters joined by two diagonal lines of letters; (2) Vertical gap trajectories consisted of horizontal spans of letters with two vertical displacements in horizontally adjacent columns; (3) Horizontal gap trajectories consisted of horizontal spans with a central portion skipped; (4) Max row trajectories consisted of a single horizontal span covering an entire row; and (5) Max Accuracy trajectories placed each letter in the location in each column with the maximum letter recognition accuracy. Of note, the max accuracy trajectory is not bounded by the number of unusual features and often results in a large number of vertical displacements or diagonal segments. If unusual features of text placement negatively impact word reading, this condition should exhibit poorer performance despite having the highest trigram letter recognition accuracy. We also tested trajectory (6) Control, consisting of the original horizontal letter placement without simulated CFL. Other trajectory types (e.g., a diagonal line down and to the right followed by a vertical gap back up to the midline) are possible but were not investigated here.

### Customizing letter trajectories: Recursive search algorithm

The heat maps for the three differently shaped simulated CFL from Experiment 1 were used to create trajectories with the different combinations of unusual features listed above. A search algorithm was used to consider trajectories that contained the specified number and type of features in each archetypal trajectory and select the one that contained the highest summed letter recognition accuracy. The search algorithm traversed the heat map from left to right column. While traversing the grid, the algorithm recursively selected each possible letter location in the next column. This brute force approach considered every possible trajectory through the grid. To reduce processing time, branches of the search were terminated if they reached a location with particularly bad letter recognition (e.g., simulated CFL) or if the trajectory exceeded the allowable number of unusual features. The same algorithm was used for each of the shaped simulated CFL heat maps (with a slight change in calculating summated letter recognition for the horizontal gap archetype) creating customized trajectories for each of the shapes of the simulated CFL.

### Methods

#### Participants

Participants were 33 students, staff members, or faculty of University of Minnesota, that participated for either course credit or cash compensation (25 females, eight males; Age mean = 24.8 years, sd = 10.7; including three participants from Experiment 1 and author SE). All participants reported normal or corrected-to-normal vision and being native English speakers. Each participant was randomly assigned to one of the three shaped simulated CFL conditions. Data from three participants were excluded from analyses (see data analysis); the final sample contained data from 30 participants.

#### Stimuli and apparatus

The apparatus, font, grid, fixation monitoring procedures, and eye tracker were the same as used in Experiment 1.

##### Word lists

Words used for testing ranged from three to 10 characters in length and were selected from the Corpus of Contemporary American English (Davies, 2009) of the 5000 most used words. Seven lists of 51 words were created that did not significantly differ in mean word frequency from each other (see Appendix A for an example word list). The number of words of each length within each list were matched and were selected to match the frequency of each word length across the corpus of words. For example, four- and five-lettered words were more frequently occurring across the corpus of words than three-lettered words, and so, each list of matched words contained more four- and five-lettered words than three-lettered words. The final constraint was that within each list, every group of n-lettered words contained a number of words divisible by three, so that equal numbers of words of each length could be tested at each of three testing positions within the trajectory, as described below.

#### Procedure

Consent, instructions, and eye tracker calibration and recalibration procedures were the same as Experiment 1 with the following additions. During instructions, participants were asked to type the words that they saw during the trial. They were asked to focus on spelling the word correctly and to correct any typos.

##### Trial structure

**Figure 4.**
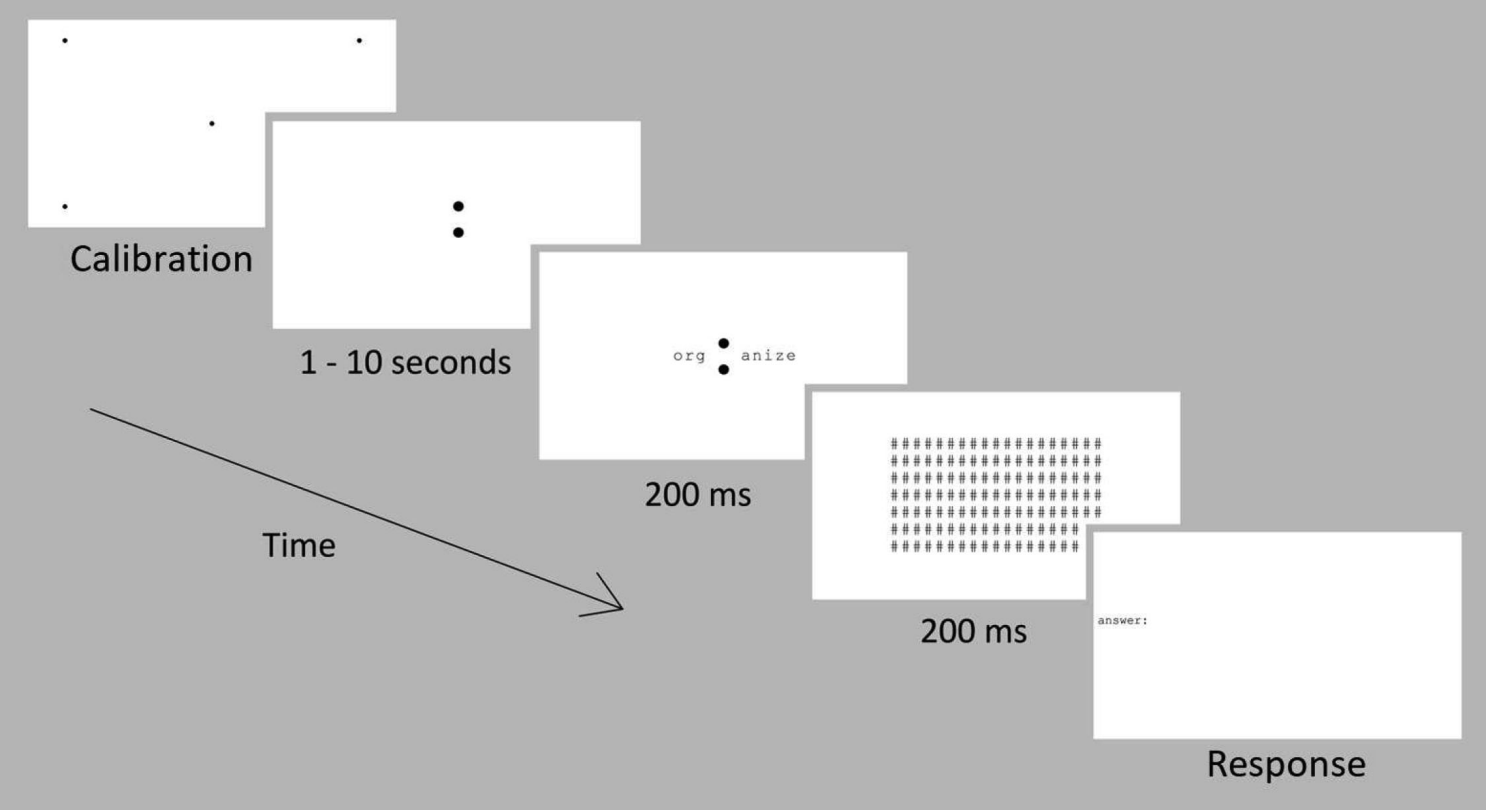
Trial structure for word recognition task in Experiment 2.

The trial structure (see Figure 4) was the same as Experiment 1 with two differences: (1) Words were shown along the tested trajectories rather than the letter trigrams used in Experiment 1; and (2) A backwards mask was presented after the word—the mask consisted of the symbol ‘#’ presented at every possible letter location. In addition, only one CFL shape was tested per participant, with condition assigned to participants in a pseudorandom sequence.

##### Practice trials

There were 18 practice trials before the experimental trials; 18 words from one list of words not used during experimental trials were used during practice trials. Participants completed three practice trials for each of the six tested remapping conditions. Each group of three trials began with a preview screen showing the to-be-tested trajectory. The experimenter remained in the room, and participants could ask any clarifying questions.

##### Experimental trials

There were 306 experimental trials, with 51 words tested from one word list for each of the six remapping conditions; words within each list were tested in random order. Trials were grouped by the remapping tested. First, 10 trials from a randomly selected trajectory were tested, and this was repeated for each trajectory until each trajectory had the first 10 trials completed. This process was repeated throughout the experiment (the final groups of trials had 11 trials each). Each grouping of 10 (or 11) trials began with a preview screen to alert participants where the letters could appear. Participants were offered to recalibrate the eye tracker between any trials following the format of Experiment 1.

### Data analysis

Data from an initial sample of 30 participants was analyzed. First, the number of trials that were lost to eye movements during the word presentation or eye tracker miscalibration were identified for each participant. Trials could not be repeated once the word was shown as participants would then have multiple presentations and the word may be primed on the repeated trial. The number of trials lost to eye movements or eye tracker miscalibrations was below 25% for each participant (mean = 8.05%).

Word recognition accuracy was calculated for each remapping strategy as the percentage of trials where the presented word was correctly typed. An outlier analysis was then performed on the word recognition accuracy for the control condition. Data from three participants were excluded for having mean word recognition on the control trials of more than 2 standard deviations below the sample mean. The control trials were the same for all CFL shapes and consisted of words presented along the middle row (no remapping or CFL). After the exclusion of data from these three participants, an additional three participants were recruited and participated using the same CFL shapes as the excluded participants. The data from these participants were not excluded using the same criteria as above. The final sample contained data from 30 participants; 10 participated in each of the three CFL shapes.

To test whether the utility of the remapping strategy differed for different CFL shapes, a mixed two-way analysis of variance was conducted on word recognition accuracy with a between-participants factor of CFL (circle, horizontal, vertical) and within-participants factor of remapping strategy (control, max row, diagonals, vertical gap, horizontal gap, max accuracy). Assumptions of sphericity were violated for the main effect of remapping strategy, χ^2^(14) = 48.505; *p* < 0.001, and Greenhouse-Geisser corrections (ε = 0.543) were made to degrees of freedom where appropriate.

### Results and discussion

#### Remapping strategies

**Figure 5.**
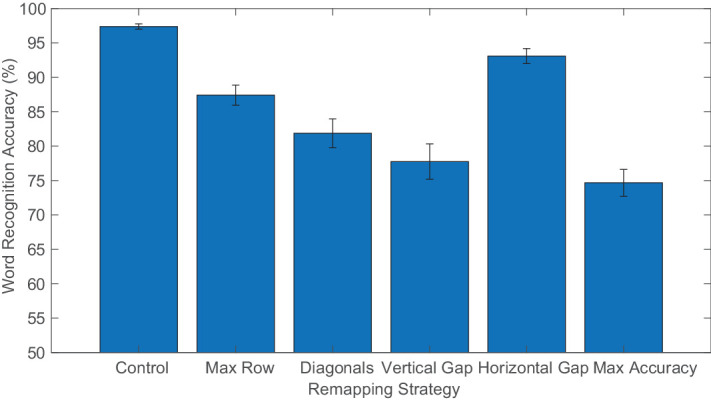
Word recognition accuracy for each of the remapping strategies averaged across all CFL shapes. Error bars represent standard error of the mean.

Remapping strategies differed in their overall effectiveness (Figure 5). There was a significant effect of remapping strategy on word recognition accuracy, *F*(2.713, 73.238) = 47.017; *p* < 0.001; $\eta_{p}^{2}$ = 0.635, and post-hoc comparisons revealed that the horizontal gap remapping resulted in the best word recognition performance (excluding the no-remapping control condition), and max accuracy the worst. The other remappings, in order of decreasing performance level, were max row, diagonals, and vertical gap, and performance between these remappings, considered pairwise, differed significantly from each other (for Holm-corrected p-values and associated statistics see Appendix B). Word recognition averaged across the remapping strategies for the differently shaped CFL was about the same; no significant effect of CFL shape was found, *F*(2,27) = 1.077; *p* > 0.05. The three CFL were roughly equal in size.

Each of the remapping strategies was customized differently for the shape of the CFL but maintained the same unusual features. As expected, control trials (with no CFL or remapping) resulted in the best word recognition performance. The horizontal gap remapping resulted in very good performance that did not differ significantly from control trials. This was unexpected because the horizontal gap remapping has an unusual feature, but it did not greatly impede word recognition. The max row remapping was the next best performing remapping. All three of these conditions featured letters that were horizontally aligned.

The diagonals and vertical gap remapping resulted in overall worse performance. These remappings each contained two unusual features. In the case of the diagonals remapping strategy, there are two diagonal lines, one rising above the midline and another dipping below the midline (in either order). The vertical gap contains two vertical shifts, one up and one down (in either order). The decreased performance in these conditions suggests that each unusual feature produces additional difficulty.

Consistent with this possibility, the max accuracy remapping strategy resulted in the worst performance. These remappings often contain many unusual features (vertical gaps and diagonal patterns) with letters jumping above and below the simulated CFL. Despite letters being placed in the locations of the grid with the best letter recognition, word recognition was greatly impeded by the presence of the unusual features.

#### Remapping strategies by CFL condition

**Figure 6.**
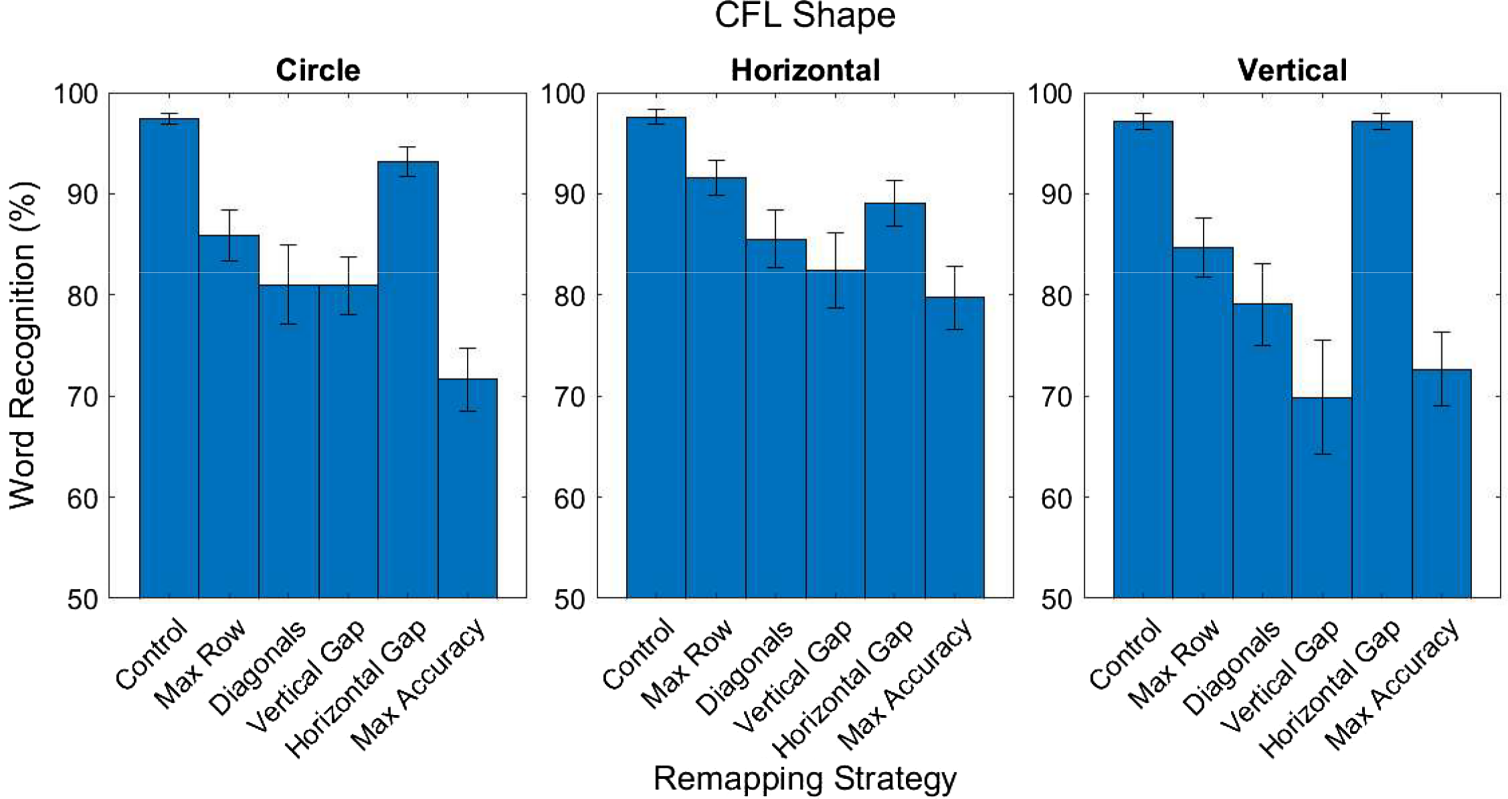
Word recognition accuracy for each of the remapping strategies for each of the simulated CFL shapes. Error bars represent standard error of the mean.

Although the horizontal gap remapping resulted in the best overall word recognition, a central aim was to assess whether word recognition resulting from different remappings was affected by differently shaped CFL. In support of the customization hypothesis, the utility of each remapping strategy on word recognition varied as a function of the shape of the CFL: The analysis of variance revealed a significant interaction between remapping strategy and CFL condition, *F*(5.425, 73.238) = 3.201; *p* = 0.01; $\eta_{p}^{2}$ = 0.192 (for Holm-corrected *p* values and associated statistics see Appendix C). Figure 6 shows word recognition performance for each remapping strategy across the three simulated CFL shapes. Across the differently shaped CFL, the horizontal gap remapping elicited the best word recognition accuracy, although performance was not significantly different from the max row remapping for the horizontal CFL.

**Figure 7.**
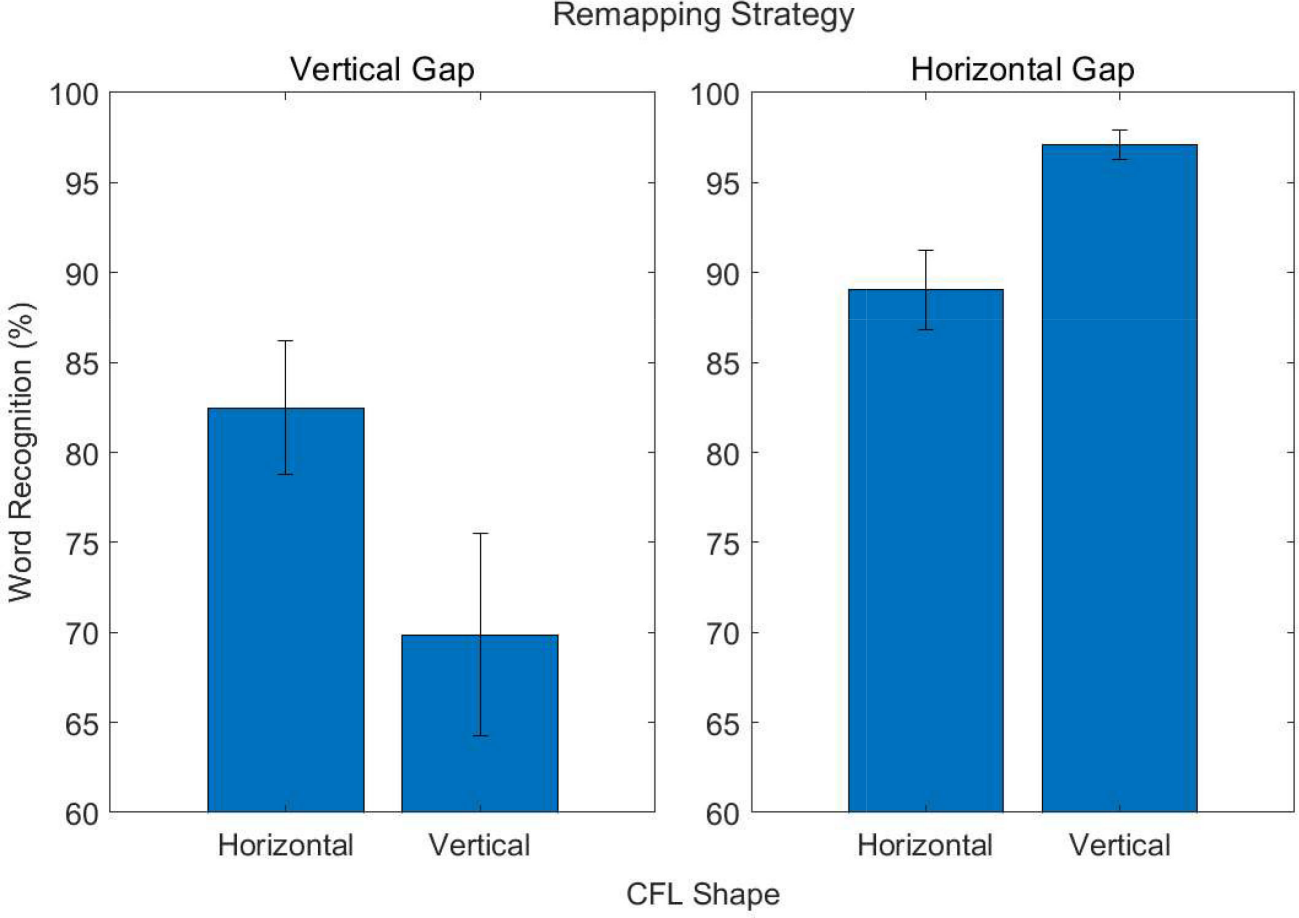
Interaction of word recognition accuracy for the horizontal and vertical gap remapping strategies for the horizontally- and vertically-elongated CFL. Error bars represent standard error of the mean.

To more clearly demonstrate the customization hypothesis a more focused analysis compared the vertical and horizontal gap trajectories for the horizontal and vertical CFL. We hypothesized that the horizontal gap trajectory would aid reading the most in the presence of vertically-elongated CFL, since a relatively small gap (three letters) was needed to bypass the CFL, and would be less beneficial for the horizontally-elongated CFL with a larger gap (five letters). Conversely, the vertical gap remapping should aid reading the most in the presence of horizontally-elongated CFL, which had a relatively small gap and should aid reading less for the vertically-elongated CFL containing a relatively large one. These predictions were borne out in the data (Figure 7). The interaction between CFL shape and trajectory type was found to be significant, *F*(1,18) = 9.348; *p* < 0.001; $\eta_{p}^{2}$ = 0.342. Although this example is used to demonstrate the customization hypothesis, the horizontal gap remapping elicited better performance for each CFL shape. Nevertheless, the relative differences in word recognition utility elicited from remappings in the presence of differently shaped CFL suggest the possibility that it may be worth customizing remappings to individuals’ unique CFL.

#### Relation to letter recognition

To examine how the trigram letter recognition accuracy related to word recognition in Experiment 2 (see Table 1), we compared average performance for each trajectory in Experiment 2 to trigram letter recognition accuracy from Experiment 1 averaged along the locations specified by each trajectory. The control remapping was excluded because it was identical across the different CFL conditions. The remaining 15 trajectories (3 CFL shapes × 5 remapping strategies) were included. The correlation failed to reach significance, *r* = −0.34, *p* > 0.10, indicating that, at least for these remappings that were selected based on having high mean trigram letter recognition accuracy, there was not a linear relationship between word and trigram letter recognition. Past studies have found that in normal reading, letter recognition performance strongly predicts reading performance (Bouwhuis & Bouma, 1979). The lack of correlation here further suggests that the presence of unusual features strongly influences word recognition, letter recognition, or both.

**Table 1.** Average letter recognition and word recognition accuracies of different remapping trajectories for each CFL shape. *Notes:* Average letter recognition accuracy was calculated from the chosen letter locations from the corresponding heatmaps in Experiment 1, and average word recognition accuracy was from Experiment 2.

	Letter recognition accuracy			Word recognition accuracy		
Remapping	Circle	Horizontal	Vertical	Circle	Horizontal	Vertical
Control	90.97	90.97	90.97	97.37	97.59	97.17
Max row	83.48	83.40	75.82	85.90	91.64	84.71
Diagonals	92.42	89.81	88.05	81.00	85.54	79.07
Vertical gap	91.36	91.05	89.34	80.92	82.49	69.88
Horizontal gap	87.08	89.65	89.82	93.14	89.03	97.11
Max accuracy	92.88	92.01	90.50	71.62	79.72	72.68

Because Experiment 1 only measured letter recognition along horizontal trajectories, we cannot tell how it was affected by the unusual features. The lack of a significant correlation is interpreted as there being a strong effect of unusual features in the presence of all remappings having strong trigram letter recognition accuracy. It is possible, even likely, that the unusual features affected letter recognition, as well as word recognition, and that measuring letter recognition along the remapped trajectories would yield better prediction of word recognition. This is an interesting direction for future work. Of course, even this measure would not capture effects of word semantics on letter recognition, which would require the use of words themselves to measure letter recognition.

## General discussion

Spatial remapping is a method of moving visual stimuli from areas of the visual field with poorer letter recognition to areas with better letter recognition. It is a promising direction for assistive devices that may prove useful in helping patients with CFL with reading (Gupta et al., 2018; Ho et al., 1995). Here, our goal was to test the value of customizing remapping to the pattern of visual field loss. Several different remapping strategies were created based on trigram letter recognition maps measured in the presence of three differently shaped simulated CFL. Our results showed that the horizontal gap remapping performed very well and elicited the best word recognition for each CFL shape (horizontal gap and max row remappings did not result in significantly different word recognition for the horizontal CFL). The horizontal gap remapping may be a promising remapping in further research on reading in other formats such as sentences or free reading. Results also showed that the utility of each remapping differed with the different CFL shapes, suggesting that customizing remappings to individual CFL may be beneficial.

Many studies have characterized the general quality of peripheral letter recognition across the horizontal meridian or at different eccentricities (Legge et al., 2001; Legge et al., 2007; McConkie & Rayner, 1975; Pelli et al., 2007; Shamsi, Chen, Liu, Pergher, & Kwon, 2021). Our results are in general agreement with this past work. Our method, however, has a different goal, more similar to perimetry: The creation of maps for individuals to guide and personalize treatment/assistance.

The resulting maps were used as inputs to a search algorithm to create remapping trajectories. The method incorporates locations with good letter recognition (rather than only avoiding locations with poor recognition occluded by the CFL), which may give it an advantage over past remapping approaches. The remapping strategies used here also preserve letter shape without producing distortions of letter shape. The precise value of these characteristics is an important direction for future research.

Overall differences between remapping strategies suggest that the unusual features had different costs associated with them. The horizontal gap strategy performed very well across the differently shaped CFL. One explanation for its benefit may be that the horizontal gap only contained one unusual feature, whereas the other strategies contained multiple. Another explanation is that deviations from horizontal arrangements of letters may be very costly to performance without extensive training (Calabrèse et al., 2017; Subramanian, Legge, Wagoner, & Yu, 2014; Yu et al., 2010). The max row remapping strategy also had horizontal continuity but also contained a vertical displacement above or below the CFL, which likely produced an additional cost to performance. The diagonals, the vertical gap, and max accuracy remapping strategies exhibited the worst word recognition performance. These remapping strategies all contained letters on different rows, a feature not present in the max row or horizontal gap strategies, or in the control condition.

The unusual features may also disrupt holistic processing of the words and this may be amplified for unusual features with horizontal discontinuity. It has been hypothesized that spatial properties of words, such as shape, may facilitate processing of words beyond letter recognition (Beech & Mayall, 2005; Healy & Cunningham, 1992; Paap, Newsome, & Noel, 1984; Perea & Rosa, 2002). Insofar as spatial configurations of letters in words aid processing, remapping of the individual letters will impede those contributions.

### Limitations and future directions

Word recognition performance using the remappings was generally good, but the absolute benefit of remapping—compared to no remapping—was not measured because some words would have been fully occluded by the simulated CFL. The prior literature has shown benefits to reading between remapped conditions and control conditions without remapping (Gupta et al., 2018; Ho et al., 1995; Massof et al., 1994; Wensveen et al., 1995). Although a no remapping condition was not included in the current design, future work extending the current approach to sentence reading should include one. The current work investigates remapping in the context of letters and words, and future work should extend this to reading sentences with eye movements in a more naturalistic setting. Just as the demands of word reading are an important factor above and beyond individual letter recognition (as demonstrated, for example, by poor performance in the max accuracy condition), it is possible that trajectories that are optimal for sentence reading differ from the ones that proved best here. The current paradigm may best predict performance in an RSVP task, and that is known to differ from more dynamic reading (Akthar, Harvey, Subramanian, Liversedge, & Walker, 2021). Nevertheless, the single word condition serves to demonstrate the utility of different remappings that depend upon properties of the scotoma.

In the current study, displays were static and eye movements forbidden; trials were aborted if fixation drifted from the center of the screen. Thus letter and word recognition as measured in this study is not representative of dynamic reading involving multiple words and eye movements. Indeed, the current study assessed a narrow instantiation of reading whereby participants are afforded an amount of time similar to that of a single fixation. The importance of eye movements in reading (e.g., Rayner, 1998) is well documented, and future work should probe how reading with spatial remapping strategies operate in the context of more typical reading with eye movements. This work may include a training period to allow the development of oculomotor strategies (e.g., Kwon et al., 2013). In addition to eye movements, this method of presenting letters and words as static displays relies on the viewer maintaining fixation at a central location. Maintaining stable fixation can be difficult for people with AMD (Macedo, Crossland & Rubin, 2011), and this should be considered when developing aids.

One final consideration was the shapes of the simulated CFL. The shapes were restricted by the underlying (invisible) letter location grids, and this made the vertical CFL more elongated than the horizontal CFL. It is likely that word recognition using the horizontal gap remapping strategy would fare worse if wider simulated CFL were tested.

An important contribution of the current study is the customization of remapping trajectories based on letter recognition maps. In the current study, letter recognition maps were averaged over eight typically-sighted participants to maximize their accuracy. When applying this mapping approach to patients with CFL, letter recognition performance across the visual field will vary dramatically across individuals. Future work will aim to replicate the creation and testing of remapping trajectories using individual letter recognition maps from each patient. These *personalized* remappings will reflect the characteristics of patients’ scotomata. The algorithm that customizes trajectories is not restricted to the CFL shapes tested here.

## Conclusions

Spatial remapping of letters can potentially aid reading in patients with CFL. Here we introduced a method to assess letter recognition across the central field and a method to use those maps to create customized spatial remappings that used different strategies for shifting letters. The horizontal gap remapping resulted in the best word recognition making it a promising remapping to test in further reading tasks. Testing word reading with these remappings also supported the customization hypothesis; the utility of remapping strategies depend on the spatial characteristics of the CFL.

## Appendix A Appendix A: One of the seven word lists used in Experiment 2

Words and their statistics are selected using the corpus of contemporary American English (Davies, 2009), accessed from www.wordfrequency.info.

**Table:**

Word	Frequency	Dispersion	Length (letters)
Dry	23809	0.94	3
Fee	20263	0.93	3
Era	19209	0.95	3
Mean	28991	0.85	4
Wood	31801	0.93	4
User	20681	0.91	4
Fast	35474	0.95	4
Hope	33019	0.98	4
Once	32180	0.97	4
Wave	26844	0.96	4
Shut	24773	0.92	4
Earn	29560	0.94	4
Fully	24842	0.96	5
Terms	30996	0.92	5
Rural	18665	0.91	5
Score	39294	0.89	5
Cloud	19214	0.92	5
Broad	27191	0.94	5
Works	22722	0.93	5
Guest	29328	0.95	5
Under	21521	0.97	5
Notion	21801	0.93	6
Demand	30519	0.93	6
Extend	25969	0.95	6
Return	31058	0.95	6
Injury	23935	0.94	6
Winner	19216	0.92	6
Pursue	20136	0.96	6
Bottom	24653	0.95	6
Advice	21970	0.96	6
Winner	19216	0.92	6
Pursue	20136	0.96	6
Bottom	24653	0.95	6
Advice	21970	0.96	6
Surface	39367	0.94	7
Collect	28946	0.95	7
Machine	38407	0.96	7
Opening	18058	0.97	7
Kitchen	39332	0.91	7
Vehicle	29421	0.96	7
Reality	38397	0.95	7
Account	19403	0.92	7
Strange	23744	0.93	7
Involved	33428	0.95	8
Capacity	21102	0.92	8
Academic	28216	0.86	8
Learning	28869	0.85	8
Supposed	31005	0.95	8
Regional	23182	0.91	8
Afternoon	33202	0.94	9
Beautiful	40052	0.94	9
Wonderful	27494	0.93	9
Appreciate	20806	0.95	10
Restaurant	35898	0.93	10
Confidence	18955	0.97	10

## Appendix B Appendix B: Pairwise comparisons of remapping strategies

*Notes: p* value adjusted for comparing a family of 15. Results are averaged over the levels of CFL Shape.

**Table:**

	Mean difference	SE	t	p_holm_
Control				
Max row	0.100	0.018	5.450	<0.001
Diagonals	0.155	0.018	8.485	<0.001
Vertical gap	0.196	0.018	10.733	<0.001
Horizontal gap	0.043	0.018	2.343	0.062
Max accuracy	0.227	0.018	12.423	<0.001
Max row				
Diagonals	0.055	0.018	3.036	0.012
Vertical gap	0.097	0.018	5.283	<0.001
Horizontal gap	−0.057	0.018	−3.106	0.012
Max accuracy	0.127	0.018	6.973	<0.001
Diagonals				
Vertical gap	0.041	0.018	2.248	0.062
Horizontal gap	−0.112	0.018	−6.142	<0.001
Max accuracy	0.072	0.018	3.937	<0.001
Vertical gap				
Horizontal gap	−0.153	0.018	−8.390	<0.001
Max accuracy	0.031	0.018	1.690	0.093
Horizontal gap				
Max accuracy	0.184	0.018	10.080	<0.001

## Appendix C Appendix C: Pairwise comparisons of interaction between remapping strategies and CFL shape

*Note: p* value adjusted for comparing a family of 153.

**Table:**

	Mean difference	SE	t	p_holm_
Circle, control				
Horizontal, control	−0.002	0.041	−0.055	1.000
Vertical, control	0.002	0.041	0.048	1.000
Circle, max row	0.115	0.032	3.623	0.043
Horizontal, max row	0.057	0.041	1.395	1.000
Vertical, max row	0.127	0.041	3.082	0.255
Circle, diagonals	0.164	0.032	5.172	<0.001
Horizontal, diagonals	0.118	0.041	2.880	0.424
Vertical, diagonals	0.183	0.041	4.456	0.003
Circle, vertical gap	0.165	0.032	5.197	<0.001
Horizontal, vertical gap	0.149	0.041	3.624	0.050
Vertical, vertical gap	0.275	0.041	6.694	<0.001
Circle, horizontal gap	0.042	0.032	1.335	1.000
Horizontal, horizontal gap	0.083	0.041	2.030	1.000
Vertical, horizontal gap	0.003	0.041	0.063	1.000
Circle, max accuracy	0.257	0.032	8.133	<0.001
Horizontal, max accuracy	0.177	0.041	4.298	0.005
Vertical, max accuracy	0.247	0.041	6.013	<0.001
Horizontal, control				
Vertical, control	0.004	0.041	0.102	1.000
Circle, max row	0.117	0.041	2.847	0.460
Horizontal, max row	0.060	0.032	1.881	1.000
Vertical, max row	0.129	0.041	3.137	0.220
Circle, diagonals	0.166	0.041	4.042	0.013
Horizontal, diagonals	0.121	0.032	3.807	0.023
Vertical, diagonals	0.185	0.041	4.511	0.002
Circle, vertical gap	0.167	0.041	4.061	0.013
Horizontal, vertical gap	0.151	0.032	4.772	<0.001
Vertical, vertical gap	0.277	0.041	6.748	<0.001
Circle, horizontal gap	0.045	0.041	1.084	1.000
Horizontal, horizontal gap	0.086	0.032	2.704	0.612
Vertical, horizontal gap	0.005	0.041	0.118	1.000
Circle, max accuracy	0.260	0.041	6.324	<0.001
Horizontal, max accuracy	0.179	0.032	5.646	<0.001
Vertical, max accuracy	0.249	0.041	6.067	<0.001
Vertical, control				
Circle, max row	0.113	0.041	2.745	0.593
Horizontal, max row	0.055	0.041	1.347	1.000
Vertical, max row	0.125	0.032	3.936	0.016
Circle, diagonals	0.162	0.041	3.939	0.018
Horizontal, diagonals	0.116	0.041	2.832	0.475
Vertical, diagonals	0.181	0.032	5.719	<0.001
Circle, vertical gap	0.163	0.041	3.959	0.017
Horizontal, vertical gap	0.147	0.041	3.576	0.058
Vertical, vertical gap	0.273	0.032	8.621	<0.001
Circle, horizontal gap	0.040	0.041	0.981	1.000
Horizontal, horizontal gap	0.081	0.041	1.982	1.000
Vertical, horizontal gap	< .001	0.032	0.020	1.000
Circle, max accuracy	0.256	0.041	6.222	<0.001
Horizontal, max accuracy	0.175	0.041	4.250	0.006
Vertical, max accuracy	0.245	0.032	7.738	<0.001
Circle, max row				
Horizontal, max row	−0.057	0.041	−1.397	1.000
Vertical, max row	0.012	0.041	0.289	1.000
Circle, diagonals	0.049	0.032	1.549	1.000
Horizontal, diagonals	0.004	0.041	0.087	1.000
Vertical, diagonals	0.068	0.041	1.664	1.000
Circle, vertical gap	0.050	0.032	1.575	1.000
Horizontal, vertical gap	0.034	0.041	0.832	1.000
Vertical, vertical gap	0.160	0.041	3.901	0.020
Circle, horizontal gap	−0.072	0.032	−2.288	1.000
Horizontal, horizontal gap	−0.031	0.041	−0.763	1.000
Vertical, horizontal gap	−0.112	0.041	−2.729	0.612
Circle, max accuracy	0.143	0.032	4.511	0.002
Horizontal, max accuracy	0.062	0.041	1.505	1.000
Vertical, max accuracy	0.132	0.041	3.220	0.174
Horizontal, max row				
Vertical, max row	0.069	0.041	1.687	1.000
Circle, diagonals	0.106	0.041	2.592	0.819
Horizontal, diagonals	0.061	0.032	1.926	1.000
Vertical, diagonals	0.126	0.041	3.061	0.265
Circle, vertical gap	0.107	0.041	2.611	0.794
Horizontal, vertical gap	0.092	0.032	2.891	0.385
Vertical, vertical gap	0.218	0.041	5.298	<0.001
Circle, horizontal gap	−0.015	0.041	−0.366	1.000
Horizontal, horizontal gap	0.026	0.032	0.823	1.000
Vertical, horizontal gap	−0.055	0.041	−1.332	1.000
Circle, max accuracy	0.200	0.041	4.874	<0.001
Horizontal, max accuracy	0.119	0.032	3.765	0.026
Vertical, max accuracy	0.190	0.041	4.618	0.002
Vertical, max row				
Circle, diagonals	0.037	0.041	0.905	1.000
Horizontal, diagonals	−0.008	0.041	−0.202	1.000
Vertical, diagonals	0.056	0.032	1.783	1.000
Circle, vertical gap	0.038	0.041	0.925	1.000
Horizontal, vertical gap	0.022	0.041	0.542	1.000
Vertical, vertical gap	0.148	0.032	4.685	<0.001
Circle, horizontal gap	−0.084	0.041	−2.053	1.000
Horizontal, horizontal gap	−0.043	0.041	−1.052	1.000
Vertical, horizontal gap	−0.124	0.032	−3.916	0.017
Circle, max accuracy	0.131	0.041	3.188	0.190
Horizontal, max accuracy	0.050	0.041	1.216	1.000
Vertical, max accuracy	0.120	0.032	3.802	0.023
Circle, diagonals				
Horizontal, diagonals	−0.045	0.041	−1.107	1.000
Vertical, diagonals	0.019	0.041	0.470	1.000
Circle, vertical gap	< .001	0.032	0.025	1.000
Horizontal, vertical gap	−0.015	0.041	−0.363	1.000
Vertical, vertical gap	0.111	0.041	2.707	0.635
Circle, horizontal gap	−0.121	0.032	−3.837	0.021
Horizontal, horizontal gap	−0.080	0.041	−1.957	1.000
Vertical, horizontal gap	−0.161	0.041	−3.924	0.019
Circle, max accuracy	0.094	0.032	2.961	0.326
Horizontal, max accuracy	0.013	0.041	0.311	1.000
Vertical, max accuracy	0.083	0.041	2.026	1.000
Horizontal, diagonals				
Vertical, diagonals	0.065	0.041	1.577	1.000
Circle, vertical gap	0.046	0.041	1.127	1.000
Horizontal, vertical gap	0.031	0.032	0.966	1.000
Vertical, vertical gap	0.157	0.041	3.814	0.026
Circle, horizontal gap	−0.076	0.041	−1.851	1.000
Horizontal, horizontal gap	−0.035	0.032	−1.103	1.000
Vertical, horizontal gap	−0.116	0.041	−2.817	0.490
Circle, max accuracy	0.139	0.041	3.390	0.103
Horizontal, max accuracy	0.058	0.032	1.840	1.000
Vertical, max accuracy	0.129	0.041	3.133	0.220
Vertical, diagonals				
Circle, vertical gap	−0.018	0.041	−0.450	1.000
Horizontal, vertical gap	−0.034	0.041	−0.832	1.000
Vertical, vertical gap	0.092	0.032	2.902	0.377
Circle, horizontal gap	−0.141	0.041	−3.427	0.093
Horizontal, horizontal gap	−0.100	0.041	−2.427	1.000
Vertical, horizontal gap	−0.180	0.032	−5.699	<0.001
Circle, max accuracy	0.074	0.041	1.813	1.000
Horizontal, max accuracy	−0.007	0.041	−0.158	1.000
Vertical, max accuracy	0.064	0.032	2.019	1.000
Circle, vertical gap				
Horizontal, vertical gap	−0.016	0.041	−0.382	1.000
Vertical, vertical gap	0.110	0.041	2.687	0.662
Circle, horizontal gap	−0.122	0.032	−3.862	0.019
Horizontal, horizontal gap	−0.081	0.041	−1.977	1.000
Vertical, horizontal gap	−0.162	0.041	−3.943	0.018
Circle, max accuracy	0.093	0.032	2.936	0.344
Horizontal, max accuracy	0.012	0.041	0.291	1.000
Vertical, max accuracy	0.082	0.041	2.006	1.000
Horizontal, vertical gap				
Vertical, vertical gap	0.126	0.041	3.070	0.262
Circle, horizontal gap	−0.107	0.041	−2.595	0.819
Horizontal, horizontal gap	−0.065	0.032	−2.068	1.000
Vertical, horizontal gap	−0.146	0.041	−3.561	0.060
Circle, max accuracy	0.109	0.041	2.646	0.733
Horizontal, max accuracy	0.028	0.032	0.874	1.000
Vertical, max accuracy	0.098	0.041	2.389	1.000
Vertical, vertical gap				
Circle, horizontal gap	−0.233	0.041	−5.665	<0.001
Horizontal, horizontal gap	−0.192	0.041	−4.664	0.001
Vertical, horizontal gap	−0.272	0.032	−8.601	<0.001
Circle, max accuracy	−0.017	0.041	−0.424	1.000
Horizontal, max accuracy	−0.098	0.041	−2.396	1.000
Vertical, max accuracy	−0.028	0.032	−0.883	1.000
Circle, horizontal gap				
Horizontal, horizontal gap	0.041	0.041	1.001	1.000
Vertical, horizontal gap	−0.040	0.041	−0.966	1.000
Circle, max accuracy	0.215	0.032	6.798	<0.001
Horizontal, max accuracy	0.134	0.041	3.269	0.151
Vertical, max accuracy	0.205	0.041	4.984	<0.001
Horizontal, horizontal gap				
Vertical, horizontal gap	−0.081	0.041	−1.967	1.000
Circle, max accuracy	0.174	0.041	4.240	0.007
Horizontal, max accuracy	0.093	0.032	2.942	0.341
Vertical, max accuracy	0.164	0.041	3.983	0.016
Vertical, horizontal gap				
Circle, max accuracy	0.255	0.041	6.207	<0.001
Horizontal, max accuracy	0.174	0.041	4.235	0.007
Vertical, max accuracy	0.244	0.032	7.718	<0.001
Circle, max accuracy				
Horizontal, max accuracy	−0.081	0.041	−1.972	1.000
Vertical, max accuracy	−0.011	0.041	−0.257	1.000
Horizontal, max accuracy				
Vertical, max accuracy	0.070	0.041	1.715	1.000
